# Estimation of population variance under ranked set sampling method by using the ratio of supplementary information with study variable

**DOI:** 10.1038/s41598-022-24296-1

**Published:** 2022-12-08

**Authors:** Rabail Alam, Muhammad Hanif, Saman Hanif Shahbaz, Muhammad Qaiser Shahbaz

**Affiliations:** 1 Department of Statistics, National College of Business Administration & Economics, Lahore, 54500 Pakistan; 2 Department of Statistics, King Abdul-Aziz University, Jeddah, Saudi Arabia; 3 Center of Research in Molecular Medicine, The University of Lahore, Lahore, 54500 Pakistan

**Keywords:** Mathematics and computing, Applied mathematics, Statistics

## Abstract

In biological and medical research, the cost and collateral damage caused during the collection and measurement of a sample are the reasons behind a compromise on the inference with a fixed and accepted approximation error. The ranked set sampling (RSS) performs better in such scenarios, and the use of auxiliary information even enhances the performance of the estimators. In this study, two generalized classes of estimators are proposed to estimate the population variance using RSS and information of auxiliary variable. The bias and mean square errors of the proposed classes of estimators are derived up to first order of approximation. Some special cases of one of the proposed class of estimators are also considered in the presence of available population parameters. A simulation study was conducted to see the performance of the members of the proposed family by using various sample sizes. The real-life data application is done to estimate the variance of gestational age of fetuses with supplementary information. The results showed that RSS design is a more accurate method than simple random sampling, to determine the population variance of hard-to-measure or destructive sampling units.

## Introduction

In many scientific fields; such as medicine, agriculture and environmental studies; various sampling methods are used to collect the data for inferences. During research studies, many environmental and biological constraints disturb the data collection procedure such as sample size, cost per sample, and destructible sample units of the study variable. These constraints highly affect the statistical analysis and inference of the study. However, ranked set sampling (RSS) design can perform better in such scenarios. McIntyre introduced the RSS technique where he applied the scheme for average yield estimation of pasture to reduce the sampling cost^1^. Later on, Stokes suggested a classical estimator for population variance in RSS with the concept of ranking error^2^. An unbiased estimator of the variance of a population under a ranked set sample is developed and is proved better than the Stokes estimator, even in small samples ^3^. Another study was conducted to evaluate the estimation of population proportion under RSS and its respective variations^4^. The efficiency of the estimator increased when the supplementary information is used alongside the study variable because there is an existence of a correlation between the estimating variables and auxiliary variables^5^.

In literature, extensive work is performed related to ratio estimation for the population mean using RSS. A study was conducted in which the ratio estimators were developed and compared in two different designs (simple RSS & Extreme RSS)^6^. The scheme of RSS received great attention of researchers, a recent study was published on balanced and unbalanced RSS^7^. The comprehensive work related to non parametric RSS is also available^8,9^.

In literature, detailed work is available for estimation of population variance in SRS. A gap is found in literature regarding the availability of estimators for population variance under RSS. This study is a little effort to address this deficiency. We have proposed a class of generalized estimators for population variance under RSS. The mean square error and bias of the proposed class of estimators is derived up to the first degree of approximation. Several members of the proposed class are developed depending upon the availability of type of supplementary information such as mean, median, tri-mean, coefficient of variation, coefficient of correlation, coefficient of skewness, kurtosis and quartile deviation. A comparison of the mean square errors on real-life data in both sampling designs (SRS & RSS) is performed to evaluate the performance of these member estimators. Moreover, the relative efficiency of these estimators is calculated in a simulation study based upon an artificial population and various sample sizes for estimation of the population variance.

To estimate the population variance, consider a population of size N that is labelled as $E = \left( {E_{1} ,E_{2} ,E_{3} , \ldots ,E_{N} } \right),$ A sample of size $j = mn$ is drawn from $E \sim \left( {Y,X} \right)$ that has a bivariate normal distribution. The process of sampling consists of $n$ random samples, each of size $n$ that are drawn from the population and the elements of each *n*th set are ordered on the basis of auxiliary variable. The smallest observation is then measured from the first sample and the second smallest from the second sample. The process is continued in this manner until the largest observation has been measured from the *n*th set. This entire cycle is repeated *m*th time and the $x_{\left( r \right)i}$ sample unit is drawn from the *r*th order of *n*th set, out of the *i*th cycle. Let $y_{\left[ r \right]i}$ and $x_{\left( r \right)i}$ be the value of the study variable and the auxiliary variable $\left( {X,Y} \right)$ respectively, where *‘i*th’ value occurred in the ‘*m*th’ cycle, as $i = \left( {1,\ldots,m} \right)$ and the ‘*r*’ is the ordered value ranked based on auxiliary variable in ‘*n*th’ sets, as $r = \left( {1,\ldots,n} \right)$. Both samples are drawn using RSS methodology where the study variable is ranked based on an auxiliary variable. The overall averages and the variances of the ranked set sample are $\hat{\mu }_{x} = \frac{1}{mn}{\sum\limits_{i = 1}^{m} {\sum\limits_{r = 1}^{n} {x_{\left( r \right)i} } } }$, $\hat{\mu }_{y} = \frac{1}{mn}{\sum\limits_{i = 1}^{m} {\sum\limits_{r = 1}^{n} {y_{\left[ r \right]i} } } }$, $\sigma_{x}^{2} = \frac{1}{mn-1}{\sum\limits_{i = 1}^{m} {\sum\limits_{r = 1}^{n} {\left( {x_{\left( r \right)i} - \hat{\mu }_{x} } \right)^{2} } } }$ and $\sigma_{y}^{2} = \frac{1}{mn-1}{\sum\limits_{i = 1}^{m} {\sum\limits_{r = 1}^{n} {\left( {y_{\left[ r \right]i} - \hat{\mu }_{y} } \right)^{2}} } }$ respectively. The ordered means and variances are $\mu_{\left( r \right)y} = \frac{1}{N}{\sum\limits_{r = 1}^{n} {y_{\left[ r \right]} } }$, $\mu_{\left( r \right)x} = \frac{1}{n}{\sum\limits_{r = 1}^{n} {x_{\left[ r \right]} } }$, ${\text{E}}\left( {y_{\left( r \right)i} - \mu_{\left( r \right)y} } \right)^{2} = \sigma_{\left( r \right)y}^{2}$ and ${\text{E}}\left( {x_{\left( r \right)i} - \mu_{\left( r \right)x} } \right)^{2} = \sigma_{\left( r \right)x}^{2}$ respectively. The other ordered measures used in this article are ${\text{E}}\left( {y_{\left( r \right)i} - \mu_{y} } \right)^{2} \left( {x_{\left( r \right)i} - \mu_{x} } \right)^{2} = \sigma_{xy}^{2}$, $\sum\limits_{r = 1}^{n} {\tau_{x\left( r \right)}^{2} = } \sum\limits_{r = 1}^{n} {\left( {\mu_{x\left( r \right)} - \mu_{x} } \right)^{2} }$, $\sum\limits_{r = 1}^{n} {\tau_{y\left( r \right)}^{2} = } \sum\limits_{r = 1}^{n} {\left( {\mu_{y\left( r \right)} - \mu_{y} } \right)^{2} }$ and $\sum\limits_{r = 1}^{n} {\tau_{y\left( r \right)}^{2} \tau_{x\left( r \right)}^{2} = } \sum\limits_{r = 1}^{n} {\left( {\mu_{y\left( r \right)} - \mu_{y} } \right)^{2} \left( {\mu_{x\left( r \right)} - \mu_{x} } \right)^{2} }$. Suppose $e_{{s_{y}^{2} }} = \left\{ {\left( {s_{y}^{2} - S_{y}^{2} } \right)/S_{y}^{2} } \right\}$, $e_{{s_{x}^{2} }} = \left\{ {\left( {s_{x}^{2} - S_{x}^{2} } \right)/S_{x}^{2} } \right\}$

$s_{y}^{2} = \left\{ {\left( {1 + e_{{s_{y}^{2} }} } \right)S_{y}^{2} } \right\}$ and $s_{x}^{2} = \left\{ {\left( {1 + e_{{s_{x}^{2} }} } \right)S_{x}^{2} } \right\}$. The expectation of square error terms are $E\left( {e_{{s_{y}^{2} }} } \right)^{2} = \sum\limits_{r = 1}^{n} {\sigma_{y\left( r \right)}^{4} } \left( {a + h} \right) + b\sum\limits_{r = 1}^{n} {\tau_{y\left( r \right)}^{2} \sigma_{y\left( r \right)}^{2} } + c\sum\limits_{r = 1}^{n} {\tau_{y\left( r \right)} \mu_{3y\left( r \right)} } + d\sum {\sum\limits_{r < s} {\sigma_{y\left( r \right)}^{2} \sigma_{y\left( r \right)}^{2} } } - \sigma_{y}^{4} = V_{y}$

$$E\left( {e_{{s_{x}^{2} }} } \right)^{2} = \sum\limits_{r = 1}^{n} {\sigma_{x\left( r \right)}^{4} \left( {a + h} \right)} + b\sum\limits_{r = 1}^{n} {\tau_{x\left( r \right)}^{2} \sigma_{x\left( r \right)}^{2} } + c\sum\limits_{r = 1}^{n} {\tau_{x\left( r \right)} \mu_{3x\left( r \right)} } + d\sum {\sum\limits_{r < s} {\sigma_{x\left( r \right)}^{2} \sigma_{x\left( r \right)}^{2} } } - \sigma_{x}^{4} = U_{x}$$

$${\text{E}}\left( {e_{{s_{y}^{2} }} e_{{s_{x}^{2} }} } \right) = a\sum\limits_{r = 1}^{n} {\sigma_{xy}^{2} + a\sigma_{x}^{2} \sum\limits_{r = 1}^{n} {\tau_{y\left( r \right)}^{2} } + 4a\sigma_{xy} \sum\limits_{r = 1}^{n} {\tau_{x} \tau_{y} } } - a\sigma_{y}^{2} \sum\limits_{r = 1}^{n} {\tau_{x\left( r \right)}^{2} } + a\sum\limits_{r = 1}^{n} {\tau_{x\left( r \right)}^{2} \tau_{y\left( r \right)}^{2} } + b\sum\limits_{r = 1}^{n} {\tau_{y\left( r \right)}^{2} \tau_{x\left( r \right)}^{2} \sigma_{xy(r)} } - \sigma_{x}^{2} \sigma_{y}^{2} = U_{x} V_{y}$$ where $$a = \frac{m}{{\left( {mn} \right)^{2} }}$$, $$b = \frac{4m}{{\left( {mn - 1} \right)^{2} }}$$, $$c = \frac{4}{{n\left( {mn - 1} \right)}}$$, $$h = \frac{{m\left( {n - 1} \right)}}{mn - 1}$$ and $$d = 2mn\frac{{\left( {m^{2} n{}^{2} - 2mn + 3} \right)}}{{\left( {mn - 1} \right)^{2} \left( {mn} \right)^{2} }}$$.

Stokes considered the errors in judgment and suggested an estimator for ${\sigma }^{2}$; which is asymptotically unbiased and more efficient than the usual *SRS* unbiased estimator for ${\sigma }^{2}$^2^

1 $$t_{o}^{2} =\frac{1}{mn-1} \sum\limits_{i = 1}^{m} {\sum\limits_{r = 1}^{n} {\left( {X_{\left[ r \right]i} - \hat{\mu }} \right)^{2} }}$$

where $$\hat{\mu } = \frac{1}{mn}{{\sum\limits_{i} {\sum\limits_{r} {X_{\left[ r \right]i} } } }}$$.

The variance of $t_{o}^{2}$ is obtained by Stokes^2^ as

2 $${\text{var}} \left( {t_{o}^{2} } \right) = \frac{m}{{\left( {mn - 1} \right)^{2} }}\left\{ \begin{gathered} \left( {\frac{mn - 1}{{mn}}} \right)^{2} \sum\limits_{r} {\mu_{4\left[ r \right]} + 4\sum\limits_{r} {\tau_{\left[ r \right]}^{2} } \sigma_{\left[ r \right]}^{2} } + 4\left( {\frac{mn - 1}{{mn}}} \right)\sum\limits_{r} {\tau_{\left[ r \right]} \mu_{3\left[ r \right]} } \hfill \\ + \frac{4m}{{\left( {mn} \right)^{2} }}\sum\limits_{r < s} {\sum {\sigma_{\left[ r \right]}^{2} \sigma_{\left[ s \right]}^{2} } + \frac{{2(m - 1) - (mn - 1)^{2} }}{{\left( {mn} \right)^{2} }}} \sum\limits_{r} {\sigma_{\left[ r \right]}^{4} } \hfill \\ \end{gathered} \right\}$$

Hadhrami have proposed the ratio estimator for the population variance based on RSS^10^ as

3 $$t_{3}^{{}} = s_{RSSy}^{2} \frac{{S_{RSSx}^{2} }}{{s_{RSSx}^{2} }}$$

where $s_{RSSx}^{2} = t_{3}^{2}$. The MSE and bias of the above estimator are

4 $$MSE(t_{3} ) = {\text{var}} \left( {s_{y}^{2} } \right) - \left( {\frac{{s_{y}^{2} }}{{s_{x}^{2} }}} \right)^{2} {\text{var}} \left( {s_{x}^{2} } \right) - 2\left( {\frac{{s_{y}^{2} }}{{s_{x}^{2} }}} \right){\text{cov}} \left( {s_{x}^{2} ,s_{y}^{2} } \right)$$

5 $$Bias(t_{3} ) = \frac{{s_{y}^{2} }}{{\left( {s_{x}^{2} } \right)^{2} }}{\text{var}} \left( {s_{x}^{2} } \right) - \left( {\frac{1}{{s_{x}^{2} }}} \right){\text{cov}} \left( {s_{x}^{2} ,s_{y}^{2} } \right).$$

## Materials and method

In this study, it is assumed that both study variable (Y) and auxiliary variable (X) have a bivariate normal distribution with high positive correlation, say $\rho \ge 0.70$. The ranking is done on the basis of auxiliary variable as it is easily and cheaply available. The variables $x$ and $y$ are both sampled by the RSS method^1^. Here the $T = S_{y}^{2}$ estimator of population variance. The R-launguage has been used to conduct the simulation study of all the forms of estimators and to compute the relative efficiency.

### Classical generalized ratio estimator

Motivated by the members of the class of estimators^11^, we have developed a generalized ratio estimator for the finite population variance under RSS scheme as:

6 $${\text{T}}_{1} = s_{y}^{2} \left( {\frac{{S_{x}^{2} }}{{s_{x}^{2} }}} \right)^{\alpha }$$

where $\alpha$ can be $( + 1, - 1)$. If $\alpha = 1$ then we have the ratio estimator of population variance from $\left( {{\text{T}}_{1} } \right)$ and if $\alpha = - 1$ then we have the product estimator of population variance $\left( {{\text{T}}_{2} } \right)$ and when $\alpha = 0$ then it is equal to the sample variance. After the simplification and taking expectations, we have following expressions for the bias and MSE of the proposed class of estimators ${\text{T}}_{1}$

7 $${\text{E}}\left( {{\text{T}}_{1} - S_{y}^{2} } \right) = S_{y}^{2} \left[ {\left( {{\text{E}}\left( {e_{{s_{y}^{2} }} } \right) - \alpha {\text{E}}\left( {e_{{s_{x}^{2} }} } \right) + \frac{{\alpha \left( {\alpha - 1} \right)}}{2}{\text{E}}\left( {e_{{s_{x}^{2} }} } \right)^{2} - \alpha {\text{E}}\left( {e_{{s_{x}^{2} }} e_{{s_{y}^{2} }} } \right)} \right) - 1} \right].$$

The bias is

8 $${\text{E}}\left( {{\text{T}}_{1} - S_{y}^{2} } \right) = S_{y}^{2} \left[ {\frac{{\alpha \left( {\alpha - 1} \right)}}{2}{\text{U}}_{x}^{2} - \alpha {\text{U}}_{{\text{x}}} {\text{V}}_{{\text{y}}} } \right].$$

Applying expectations in Eq. (7), the mean square error is:

9 $${\text{MSE}}\left( {{\text{T}}_{{1}} } \right) = S_{y}^{4} \left[ {{\text{V}}_{y}^{2} + \alpha {\text{U}}_{x}^{2} - 2\alpha {\text{V}}_{{\text{y}}} {\text{U}}_{x} } \right].$$

### Generalized class of estimators with auxiliary information

Motivated by Singh^12^, we have proposed another generalized class of ratio estimators to estimate the finite population variance by utilizing single auxiliary information under RSS technique. The proposed estimator is:

10 $${\text{T = }}\kappa_{1} s_{y}^{2} \left\{ {\frac{{cS_{x}^{2} - ds_{x}^{2} }}{{\left( {c - d} \right)S_{x}^{2} }}} \right\}^{\lambda } + \kappa_{2} s_{y}^{2} \left\{ {\frac{{\left( {a + b} \right)S_{x}^{2} }}{{aS_{x}^{2} + bs_{x}^{2} }}} \right\}^{\delta },$$

where $\left( {\kappa_{1} ,\kappa_{2}} \right)$ and $\left( {\lambda ,\delta } \right)$ are the constants which take finite values and $\left( {a,b,c,d} \right)$ are function of known population parameters of auxiliary variable $X$, such as $\overline{X},C_{x} ,\beta_{1} \left( x \right),\beta_{2} \left( x \right)$ and $\rho_{xy}$. When values of $\left( {\kappa_{1} ,\kappa_{2} ,a,b,c,d,\lambda ,\delta } \right)$ are suitably chosen then several existing estimators can be obtained from proposed generalized class of estimators T. In addition to existing estimators, some new estimators are generated from proposed class of estimators ${\text{T}}_{i} = \left( {3,4,5,6,7,8} \right)$ which are given in Table 1.

**Table 1:** Some members of class of estimators.

Estimator	Values of constant					
	$\lambda$	$\delta$	a	b	c	d
${\text{T}}_{3} = \kappa_{1} s_{y}^{2} \left\{ {\frac{{S_{x}^{2} - \beta_{1\left( x \right)} s_{x}^{2} }}{{\left( {1 - \beta_{1\left( x \right)} } \right)S_{x}^{2} }}} \right\} + \kappa_{2} s_{y}^{2} \left\{ {\frac{{\left( {1 + T_{m} } \right)S_{x}^{2} }}{{S_{x}^{2} + T_{m} s_{x}^{2} }}} \right\}$	1	1	1	$\beta_{1\left( x \right)}$	1	$Tm$
${\text{T}}_{4} = \kappa_{1} s_{y}^{2} \left\{ {\frac{{S_{x}^{2} - \beta_{2\left( x \right)} s_{x}^{2} }}{{\left( {1 - \beta_{2\left( x \right)} } \right)S_{x}^{2} }}} \right\} + \kappa_{2} s_{y}^{2} \left\{ {\frac{{\left( {1 + C_{x} } \right)S_{x}^{2} }}{{S_{x}^{2} + C_{x} s_{x}^{2} }}} \right\}$	1	1	1	$\beta_{2\left( x \right)}$	1	$C_{x}$
${\text{T}}_{5} = \kappa_{1} s_{y}^{2} \left\{ {\frac{{\rho_{xy} S_{x}^{2} - \beta_{1\left( x \right)} s_{x}^{2} }}{{\left( {\rho_{xy} - \beta_{1\left( x \right)} } \right)S_{x}^{2} }}} \right\} + \kappa_{2} s_{y}^{2} \left\{ {\frac{{\left( {\overline{X} + \tilde{X}} \right)S_{x}^{2} }}{{\overline{X}S_{x}^{2} + \tilde{X}s_{x}^{2} }}} \right\}$	1	1	$\rho_{xy}$	$\beta_{1\left( x \right)}$	$\overline{X}$	$\tilde{X}$
${\text{T}}_{6} = \kappa_{1} s_{y}^{2} \left\{ {\frac{{\rho_{xy} S_{x}^{2} - \beta_{2\left( x \right)} s_{x}^{2} }}{{\left( {\rho_{xy} - \beta_{2\left( x \right)} } \right)S_{x}^{2} }}} \right\} + \kappa_{2} s_{y}^{2} \left\{ {\frac{{\left( {\overline{X} + Qd} \right)S_{x}^{2} }}{{\overline{X}S_{x}^{2} + Qds_{x}^{2} }}} \right\}$	1	1	$\rho_{xy}$	$\beta_{2\left( x \right)}$	$\overline{X}$	$Qd$
${\text{T}}_{7} = \kappa_{1} s_{y}^{2} \left\{ {\frac{{\rho_{xy} S_{x}^{2} - \beta_{1\left( x \right)} s_{x}^{2} }}{{\left( {\rho_{xy} - \beta_{1\left( x \right)} } \right)S_{x}^{2} }}} \right\} + \kappa_{2} s_{y}^{2} \left\{ {\frac{{\left( {Tm + \tilde{X}} \right)S_{x}^{2} }}{{TmS_{x}^{2} + \tilde{X}s_{x}^{2} }}} \right\}$	1	1	$\rho_{xy}$	$\beta_{1\left( x \right)}$	$Tm$	$\tilde{X}$
${\text{T}}_{8} = \kappa_{1} s_{y}^{2} \left\{ {\frac{{\rho_{xy} S_{x}^{2} - \beta_{2\left( x \right)} s_{x}^{2} }}{{\left( {\rho_{xy} - \beta_{2\left( x \right)} } \right)S_{x}^{2} }}} \right\} + \kappa_{2} s_{y}^{2} \left\{ {\frac{{\left( {Tm + Qd} \right)S_{x}^{2} }}{{TmS_{x}^{2} + Qds_{x}^{2} }}} \right\}$	1	1	$\rho_{xy}$	$\beta_{2\left( x \right)}$	$Tm$	$Qd$

Using error term notations in Eq. (10), we get

11 $${\text{T = }}\kappa_{1} S_{y}^{2} \left( {1 + e_{{s_{y}^{2} }} } \right)\left( {1 - \eta_{2} e_{{s_{x}^{2} }} } \right)^{\lambda } + \kappa_{2} S_{y}^{2} \left( {1 + e_{{s_{y}^{2} }} } \right)\left( {1 + \eta_{1} e_{{s_{x}^{2} }} } \right)^{\delta }$$

where $\psi_{2} = \frac{d}{{\left( {c - d} \right)}}$, $\psi_{1} = \frac{b}{{\left( {a + b} \right)}}$. Taking expectation and after simplification we have

12 $${\text{E}}\left( {{\text{T}} - S_{y}^{2} } \right){ = }S_{y}^{2} \left[ \begin{gathered} \kappa_{1} \left\{ {1 + {\text{E}}\left( {e_{{s_{y}^{2} }} } \right) - \psi_{2} \lambda {\text{E}}\left( {e_{{s_{x}^{2} }} } \right) + \frac{{\lambda \left( {\lambda - 1} \right)}}{2}\psi_{2}^{2} {\text{E}}\left( {e_{{s_{x}^{2} }} } \right)^{2} - \psi_{2} \lambda {\text{E}}\left( {e_{{s_{x}^{2} }} e_{{s_{y}^{2} }} } \right)} \right\} \hfill \\ + \kappa_{2} \left( {1 + {\text{E}}\left( {e_{{s_{y}^{2} }} } \right) - \psi_{1} \delta {\text{E}}\left( {e_{{s_{x}^{2} }} } \right) + \frac{{\delta \left( {\delta + 1} \right)}}{2}\psi_{1}^{2} {\text{E}}\left( {e_{{s_{x}^{2} }} } \right)^{2} - \psi_{1} \delta {\text{E}}\left( {e_{{s_{x}^{2} }} e_{{s_{y}^{2} }} } \right)} \right) - 1 \hfill \\ \end{gathered} \right]$$

The bias is obtained by using error notations terms from section-I (“Introduction” section) and is

13 $$B(T) = S_{y}^{2} \left[ {\kappa_{1} \left\{ {1 + \frac{{\lambda \left( {\lambda - 1} \right)}}{2}\psi_{2}^{2} {\text{U}}_{x} - \psi_{2} \lambda {\text{U}}_{{\text{x}}} {\text{V}}_{{\text{y}}} } \right\} + \kappa_{2} \left( {1 + \frac{{\delta \left( {\delta + 1} \right)}}{2}\psi_{1}^{2} {\text{U}}_{x} - \psi_{1} \delta {\text{U}}_{{\text{x}}} {\text{V}}_{{\text{y}}} } \right) - 1} \right].$$

Following expression of MSE is obtained after taking square and expectation of Eq. (12) and  ignoring the higher order terms as

14 $${\text{E}}\left( {{\text{T}} - S_{y}^{2} } \right)^{2} { = }\,\,\,S_{y}^{4} + S_{y}^{4} \left[ \begin{gathered} \kappa_{1}^{2} \left\{ {{\text{E}}\left( {e_{{s_{y}^{2} }} } \right)^{2} - \psi_{2}^{2} \lambda^{2} {\text{E}}\left( {e_{{s_{x}^{2} }} } \right)^{2} - \psi_{2} \lambda {\text{E}}\left( {e_{{s_{x}^{2} }} e_{{s_{y}^{2} }} } \right)} \right\} \hfill \\ + \kappa_{2}^{2} \left\{ {{\text{E}}\left( {e_{{s_{y}^{2} }} } \right)^{2} - \psi_{1}^{2} \delta^{2} {\text{E}}\left( {e_{{s_{x}^{2} }} } \right)^{2} - \psi_{1} \delta {\text{E}}\left( {e_{{s_{x}^{2} }} e_{{s_{y}^{2} }} } \right)} \right\} \hfill \\ + 2\kappa_{1} \kappa_{{_{2} }} \left\{ {{\text{E}}\left( {e_{{s_{y}^{2} }} } \right)^{2} - \varphi \left( {e_{{s_{x}^{2} }} } \right) - 2\varphi {\text{E}}\left( {e_{{s_{x}^{2} }} e_{{s_{y}^{2} }} } \right) + \psi_{1} \psi_{2} \lambda \delta {\text{E}}\left( {e_{{s_{x}^{2} }} } \right)^{2} } \right\} \hfill \\ - 2\kappa_{1} \left\{ {{\text{E}}\left( {e_{{s_{y}^{2} }} } \right) - \psi_{2}^{2} \lambda^{2} {\text{E}}\left( {e_{{s_{x}^{2} }} } \right) - \psi_{2} \lambda {\text{E}}\left( {e_{{s_{x}^{2} }} e_{{s_{y}^{2} }} } \right)} \right\} \hfill \\ - 2\kappa_{2} \left\{ {{\text{E}}\left( {e_{{s_{y}^{2} }} } \right) - \psi_{1}^{2} \delta^{2} {\text{E}}\left( {e_{{s_{x}^{2} }} } \right) - \psi_{1} \delta {\text{E}}\left( {e_{{s_{x}^{2} }} e_{{s_{y}^{2} }} } \right)} \right\} \hfill \\ \end{gathered} \right]$$

where $\varphi = \left( {\psi_{2} \lambda - \psi_{1} \delta } \right)$. Using notation given in section-I (Introduction) the expression of mean square error is

15 $${\text{MSE}}\left( {\text{T}} \right){ = }\,\,\,S_{y}^{4} \left[ {1 + \left( {\kappa_{1}^{2} {\text{A + }}\kappa_{2}^{2} {\text{B + }}2\kappa_{1} \kappa_{{_{2} }} C} \right) - 2\left( {\kappa_{1} D + \kappa_{2} E} \right)} \right],$$

where $${\text{A = V}}_{y}^{2} + \lambda^{2} \psi_{2}^{2} {\text{U}}_{x}^{2} - 2\psi_{2} \lambda {\text{U}}_{x} {\text{V}}_{y}$$, $${\text{B = V}}_{y}^{2} + \delta^{2} \psi_{1}^{2} {\text{U}}_{x}^{2} - 2\psi_{1} \delta {\text{U}}_{x} {\text{V}}_{y}$$$${\text{C = V}}_{y}^{2} - 2\varphi {\text{U}}_{x} {\text{V}}_{y} + \psi_{1} \psi_{2} \lambda \delta {\text{U}}_{x}^{2}$$.

$${\text{D = }}\psi_{2} \lambda {\text{U}}_{x} {\text{V}}_{y}$$, and $${\text{E = }}\psi_{1} \delta {\text{U}}_{x} {\text{V}}_{y}$$.

Differentiating Eq. (15) with respect to $\kappa_{1}$ and $\kappa_{2}$ and equating to zero, the optimum values of $\kappa_{1}^{*}$ and $\kappa_{2}^{*}$ are, respectively, obtained as

16 $$\kappa_{1} = \frac{{\left( {BD - CE} \right)}}{{\left( {AB - C^{2} } \right)}} = \kappa_{1}^{*}$$

and

17 $$\kappa_{2} = \frac{{\left( {AE - CD} \right)}}{{\left( {AB - C^{2} } \right)}} = \kappa_{2}^{*}.$$

Using the above optimum values, the minimum MSE of generalized class of estimators T is

18 $${\text{MSE}}\left( {\text{T}} \right){ = }S_{y}^{4} \left[ {1 + \left( {\kappa_{1}^{2*} {\text{A + }}\kappa_{2}^{2*} {\text{B + }}2\kappa_{1}^{*} \kappa_{2}^{*} C} \right) - 2\left( {\kappa_{1}^{*} D + \kappa_{2}^{*} E} \right)} \right].$$

From above generalized class of ratio estimators many forms can be formed on the basis of availbilty of population parameters of suplementry information. Some members of this class are given in Table 1 above.

## Results

In this section, the real-life data is used for empirical study to obtain mean square error for explaining the advantage of RSS estimators over simple random sampling (SRS). Next, the simulation study is presented with the percent relative efficiencies of various estimators.

### Applications

The RSS has an advantage in biostatistics to provide greater efficacy in small sample sizes when the variable of interest is difficult to obtain, destructive and costly. A study on the “Assessment of gestational age and weight” was done by a student in 2014–2015 in which the accuracy of gestational age was assessed by Ultrasound. A total of 400 ultrasounds were performed on pregnant ladies. From this study we have taken two highly correlated variables X = femur length of fetus and Y = gestational age. We have, then calculated the mean square error of estimators by RSS and SRS procedure shown in Table 2. From a population size of 400, we have drawn one sample of size = 12 where set size (m = 3) and no. of cycles are (n = 4) from RSS and the other sample of size of 12 is drawn by using the SRS method. The measures obtained from the population are $\mu_{x}$ = 6.1488, $\mu_{y}$ = 31.990 $\sigma_{x}^{2}$ = 0.831, $\sigma_{y}^{2}$ = 17.025, $\beta_{1\left( x \right)}$ =  − 0.223, $\beta_{2\left( x \right)}$ =  − 0.649, $\rho_{xy}$ = 0.997, $Tm_{x}$ = 6.175, $Qd_{x}$ = 0.75, $Me_{x}$ = 6.200, $\sigma_{\left( r \right)y}^{2}$ = 6.9146, $\sigma_{\left( r \right)x}^{2}$ = 0.1985.

**Table 2:** Mean square error of different estimators.

Estimators	SRS MSE	RSS MSE
${\text{T}}_{\left( 1 \right)} = s_{y}^{2} \left( {\frac{{S_{x}^{2} }}{{s_{x}^{2} }}} \right)$	25,450.88	19,152.43
${\text{T}}_{\left( 2 \right)} = s_{y}^{2} \left( {\frac{{s_{x}^{2} }}{{S_{x}^{2} }}} \right)$	25,818.66	19,412.12
${\text{T}}_{3} = \kappa_{1} s_{y}^{2} \left\{ {\frac{{S_{x}^{2} - \beta_{1\left( x \right)} s_{x}^{2} }}{{\left( {1 - \beta_{1\left( x \right)} } \right)S_{x}^{2} }}} \right\} + \kappa_{2} s_{y}^{2} \left\{ {\frac{{\left( {1 + T_{m} } \right)S_{x}^{2} }}{{S_{x}^{2} + T_{m} s_{x}^{2} }}} \right\}$	378.959	290.2143
${\text{T}}_{4} = \kappa_{1} s_{y}^{2} \left\{ {\frac{{S_{x}^{2} - \beta_{2\left( x \right)} s_{x}^{2} }}{{\left( {1 - \beta_{2\left( x \right)} } \right)S_{x}^{2} }}} \right\} + \kappa_{2} s_{y}^{2} \left\{ {\frac{{\left( {1 + C_{x} } \right)S_{x}^{2} }}{{S_{x}^{2} + C_{x} s_{x}^{2} }}} \right\}$	960.973	204.0161
${\text{T}}_{5} = \kappa_{1} s_{y}^{2} \left\{ {\frac{{\rho_{xy} S_{x}^{2} - \beta_{1\left( x \right)} s_{x}^{2} }}{{\left( {\rho_{xy} - \beta_{1\left( x \right)} } \right)S_{x}^{2} }}} \right\} + \kappa_{2} s_{y}^{2} \left\{ {\frac{{\left( {\overline{X} + \tilde{X}} \right)S_{x}^{2} }}{{\overline{X}S_{x}^{2} + \tilde{X}s_{x}^{2} }}} \right\}$	286.786	259.7252
${\text{T}}_{6} = \kappa_{1} s_{y}^{2} \left\{ {\frac{{\rho_{xy} S_{x}^{2} - \beta_{2\left( x \right)} s_{x}^{2} }}{{\left( {\rho_{xy} - \beta_{2\left( x \right)} } \right)S_{x}^{2} }}} \right\} + \kappa_{2} s_{y}^{2} \left\{ {\frac{{\left( {\overline{X} + Qd} \right)S_{x}^{2} }}{{\overline{X}S_{x}^{2} + Qds_{x}^{2} }}} \right\}$	807.0286	259.5786
${\text{T}}_{7} = \kappa_{1} s_{y}^{2} \left\{ {\frac{{\rho_{xy} S_{x}^{2} - \beta_{1\left( x \right)} s_{x}^{2} }}{{\left( {\rho_{xy} - \beta_{1\left( x \right)} } \right)S_{x}^{2} }}} \right\} + \kappa_{2} s_{y}^{2} \left\{ {\frac{{\left( {Tm + \tilde{X}} \right)S_{x}^{2} }}{{TmS_{x}^{2} + \tilde{X}s_{x}^{2} }}} \right\}$	287.4439	259.7266
${\text{T}}_{8} = \kappa_{1} s_{y}^{2} \left\{ {\frac{{\rho_{xy} S_{x}^{2} - \beta_{2\left( x \right)} s_{x}^{2} }}{{\left( {\rho_{xy} - \beta_{2\left( x \right)} } \right)S_{x}^{2} }}} \right\} + \kappa_{2} s_{y}^{2} \left\{ {\frac{{\left( {Tm + Qd} \right)S_{x}^{2} }}{{TmS_{x}^{2} + Qds_{x}^{2} }}} \right\}$	264.5108	259.6234

Based on the Table 2, it is obvious that the mean square error of the RSS estimators has lower value than the SRS estimators. The estimator $T_{2}$ is a product estimator and its mean square error is near to each other in both sampling designs as the both variables have negative correlation in real-life data.

### Simulation study

The performance of the proposed estimator is compared with the existing estimator based on simulation study. The simulation study is performed by generating random observation from a normal distribution. We generated artificial population of size N = 5000 on the auxiliary variable $X$ from a normal distribution with mean 10 and standard deviation 2. Using the auxiliary variable, the study variable $Y$ was generated by using the following linear equation

$$Y_{i} = 5 + 1.87X_{i} + e_{i}$$

where $e_{i}$ is $N(0,1).$ After generating the random population artificially, both sampling techniques RSS & SRS are performed to draw two independent samples respectively and we have computed all the forms of the proposed generalized estimators in different sample sizes for comparison. The procedure is repeated for 10,000 times and using 10,000 values of each estimator, the variance of each estimator is calculated. The results are given in Table 3 below. The percent relative efficiency of estimator calculated from the simulated variance of estimators by RSS and SRS procedure. The behavior of simulated variances in RSS and SRS is shown by graph-I which contains  relative efficiency at different sample sizes.

**Table 3:** Percent relative efficiencies (PREs) of estimators at rho = 0.90.

Percentage relative efficiency					
SRS sample size	9	12	16	20	25
RSS sample size	m = 3, n = 3	m = 3, n = 4	m = 4, n = 4	m = 4, n = 5	m = 5, n = 5
T1	**2790.1020**	**2239.1620**	**2397.1550**	**2044.3430**	**2262.4710**
T2	0.0323	0.3480	0.3819	0.4154	0.4776
T3	231.1386	260.9198	231.1386	192.9843	105.1537
T4	277.3733	181.7453	135.8969	130.7218	123.0522
T5	29.2569	28.1691	28.8956	27.8468	31.4923
T6	**283.0801**	**293.5363**	**394.2184**	**422.9133**	**488.3382**
T7	18.5723	5.2971	6.0491	5.2340	6.0502
T8	8.0509	5.3677	6.9015	5.5186	5.9868

Significant values are in bold.

As shown in table 3, T1 (proposed RSS estimator) performed more than 2000 percent better than conventional SRS ratio estimator of Isaki^13^ in all sample sizes. T3,T4 and T6 (RSS estimators) also performed 200 percent better than the SRS estimators. Moreover T6 & T5 (RSS estimators) showed better performance as sample size increased, whereas the performance of T3,T4 (RSS estimators) decreased as the sample size is increased and same is the behaviour of T7 & T8. The T2 is basically product estimator and its performance depends upon the negtive correlation, that is why the RSS estimator is not a better choice than SRS estimator where correlation is negative. Uniquelly T6 performed better when set size and cycle size were equal.

**Figure 1:**
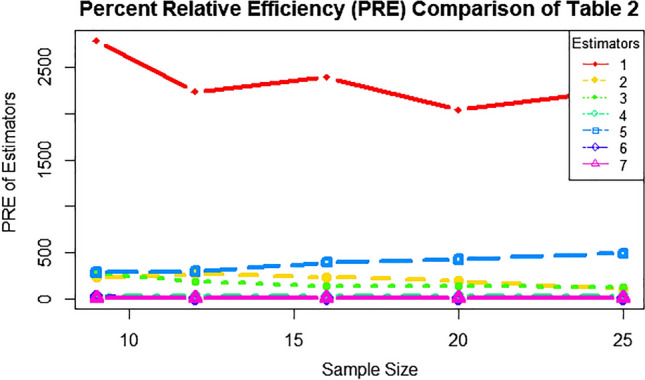
Above are the PRE of estimators with respect to different sample sizes, whereas T1, T3, T4, T5, T6, T7 and T8 are presented in red, yellow, green, aqua blue, light blue, purple and pink colors, respectively.

From this simulation study, we can conclude that all RSS estimators have greater percent relative efficiency then the SRS estimators. The red line shows that $T_{1}$ (ratio estimator) has a greater relative efficiency. The $T_{2}$ product estimator cannot be simulated as the $X$ and $Y$ are highly negative correlated variables taken from normal population (Fig 1). For the further evaluation of properties of the suggested estimators the simulation study is conducted on lower correlation in Table 4 given below:

**Table 4:** Percent relative efficiencies (PREs) of estimators at rho = 0.51.

PREs					
Sample size (SRS &RSS)	9 (n = 3, m = 3)	12 (n = 3, m = 4)	16 (n = 4, m = 4)	20 (n = 4, m = 5)	25 (n = 5, m = 5)
T1	1.04432	1.47021	1.5327	1.5351	1.8300
T2	0.61561	0.64721	0.7642	0.7336	0.7963
T3	349.984	335.604	363.683	528.781	489.1021
T4	1668.446	3682.987	1909,009	1543.30	862.6497
T5	**40,140**	**38,431**	**31,178**	**39,007**	**37,689**
T6	115.3035	110.164	107.438	126.624	134.3908
T7	1347.62	1097.36	1364.610	1388.869	1341.826
T8	**0.3898**	1.51498	7.30963	2.105681	3.09583

Significant values are in bold.

As shown in Table 4, the T1—proposed RSS estimator performed better than conventional ratio estimator of SRS. The T2 is a product estimator and its performance depends upon the negative correlation that’s why the RSS estimator is not better then SRS estimator when correlation is negative. Overall, we can see that most of the RSS estimators outperformed corresponding to SRS estimators. In particular, the estimator T5 is the best estimator as it has highest relative efficiency.

## Discussion

In this study, ratio and generalized class of estimators for population variance are suggested under RSS design utilizing one auxiliary variable. The mean square error of the estimator for population variance for RSS are obtained. We have considered real population as well as simulation data for comparison of proposed estimators with SRS design. It can be clearly observed from Table 2. that the mean square error of RSS design is giving minimum values than the simple random designs in real life population and the percent relative efficiencies of the estimators are shown in Tables 3 and 4 and are greater than the SRS design. The PREs of estimators are calculated through simulated data and using different samples. The estimator $T_{1}$, which is a ratio estimator, provides higher values in percent relative efficiencies for all the sample size as can be seen in Tables 3 and 4. The estimator $T_{6}$ is the second-best estimator with respect to percent relative efficiencies as can be seen from the Graph-I. It is shown that the ratio estimator provides higher percent relative efficiency than the other estimators because in the generalized class of estimators when the constants $\left( {\kappa_{1} ,\kappa_{2} } \right)$ provides negative value, then the behavior of ratio estimator changes to the product estimator. This will also affect the efficiency of the estimator when the population is highly positively correlated. Overall, it proved that RSS design estimators are more efficient in small-size sampling studies.

## Conclusion

The main purpose of this study is to propose a generalized class of estimators for population variance in RSS utilizing one auxiliary variable and comparing its efficiency with the corresponding estimators in SRS design. We have found that our RSS estimator is practically best estimator in situations where the study variable is costly, destructive and hard to achieve. We can achieve greater efficiency in small sample size based studies like biological sciences, medical experimental researches, environmental sciences and in engineering using the estimators proposed in this study.

## Data Availability

The authors confirm that the data supporting the findings of this study are available within the article and the programming files will available on request. Additional information/query related to this paper may be requested from the corresponding authors: Rabail Alam (rabail.alam@yahoo.com, raabail.alam@imbb.uol.edu.pk).
